# German Expert Consensus on Opioid-Induced Constipation (OIC): Recommendations and a Treatment Algorithm for Clinical Practice

**DOI:** 10.3390/jcm15062369

**Published:** 2026-03-20

**Authors:** Bastian Wobbe, Viola Andresen, Ralf Baron, Jürgen Eiche, Frank Elsner, Sven Gottschling, Jens Keßler, Hartmut Link, Philipp C. G. Müller-Schwefe, Ulf Schutter, Martin Storr, Michael A. Überall, Stefan Wirz

**Affiliations:** 1 Department of Internal Medicine—Oncology and Hematology, Klinikum Oldenburg AoR, 26133 Oldenburg, Germany; 2 Medizinicum Stephansplatz, 20354 Hamburg, Germany; 3 Division of Neurological Pain Research and Therapy, University Hospital Schleswig-Holstein, Campus Kiel, 24149 Kiel, Germany; 4 Median Heinrich Mann Klinik, 36448 Bad Liebenstein, Germany; 5 DGS Regional Center, 36433 Bad Salzungen, Germany; 6 Department of Palliative Medicine, Medical Faculty, RWTH Aachen University, 52074 Aachen, Germany; 7 Centre of Palliative Care and Pediatric Pain, Saarland University Medical Center, 66421 Homburg, Germany; 8 Pain Medicine Section, Department of Anesthesiology, Medical Faculty Heidelberg, Heidelberg University, 69120 Heidelberg, Germany; 9 Hematology and Medical Oncology, Westpfalz-Klinikum, 677655 Kaiserslautern, Germany; 10 Pain and Palliative Care Center, 73033 Göppingen, Germany; 11 DGS Regional Center, Specialist Medical Center, Multimodal Pain Therapy at Marienhospital Marl, 45768 Marl, Germany; 12 Center for Endoscopy, Internal Medicine Center Starnberg-Gauting, 82319 Starnberg, Germany; 13 Institute of Neurological Sciences, 90411 Nuremberg, Germany; 14 Department for Anesthesiology, Intensive Medicine, Pain and Palliative Medicine, Center for Pain Medicine, Weaning Center, Cura Hospital/GFO-Clinics Bonn, 53604 Bad Honnef, Germany

**Keywords:** opioid, opioid-induced constipation, laxatives, PAMORA, chronic pain

## Abstract

**Background/Objectives:** Opioid-induced constipation (OIC) is a frequent adverse effect of opioid therapy. In contrast to other opioid-related side effects, OIC usually does not improve over time and significantly impairs the quality of life of affected patients. Despite its high prevalence, OIC remains underdiagnosed and undertreated in clinical practice, which has been demonstrated in several European countries. Healthcare data indicates that approximately 2.3 million people in Germany received potentially OIC-inducing opioids in 2023, the majority being patients with chronic non-cancer pain. **Methods:** An interdisciplinary board of experts in gastroenterology, pain medicine, neurology, oncology, and palliative care developed consensus-based recommendations to improve the diagnosis and management of OIC. Fifteen statements were drafted according to current national German and international guidelines and literature and subsequently discussed. Out of the fifteen statements, twelve statements remained, which achieved consensus with at least 90% agreement. **Results:** The consensus statements address key aspects of OIC management, including pathophysiology, patient education, diagnosis, prevention, treatment and structured follow-up. Following the statements, a practical treatment algorithm was developed to facilitate clinical implementation. Use of validated tools such as the Bowel Function Index (BFI) for diagnosis and monitoring, early initiation of laxative therapy and timely escalation to mechanism-oriented therapy with peripherally acting μ-opioid receptor antagonists (PAMORAs) in cases of inadequate response have been recommended by the panel. Accordingly, treatment should follow an approach with the following steps: (1) Laxative, (2) switch to PAMORA, (3) rotation of PAMORA, and (4) combination of PAMORA with laxative. In Europe, the PAMORAs methylnaltrexone, naloxegol and naldemedine are approved for the treatment of OIC. **Conclusions:** This consensus paper provides both evidence-based and practice-oriented recommendations for the systematic management of OIC. By promoting patient education, early recognition, structured evaluation and stepwise treatment escalation, the presented statements and algorithm aim to improve patient outcomes and quality of life under opioid therapy including better adherence to opioid therapy.

## 1. Introduction

Opioid-induced constipation (OIC) is a common adverse effect of opioid therapy which, in contrast to other adverse effects of opioid use, typically does not improve over time [1,2]. Consequently, management of OIC is an important factor for the quality of life of patients undergoing long-term pain therapy. OIC is caused by the activation of peripheral opioid receptors in the gastrointestinal tract leading to reduced gut motility, decreased secretion, and increased sphincter tone [3].

OIC is widely underdiagnosed and undertreated despite its frequency and impact on patients’ quality of life [1]. A patient survey conducted in the United States, Canada, Germany, and the United Kingdom reported that a large proportion of patients are not satisfied with the management of their OIC [4]. Healthcare professionals (HCPs) demonstrated limited or incomplete awareness regarding their patients’ burden of OIC, including symptom severity, level of laxative use, and the limited benefits of laxative use [4]. These discrepancies were at least partially ascribed to insufficient communication about OIC between HCPs and patients [4]. Therefore, improvements in patient education and communication are necessary. According to a survey in five European countries (France, Germany, Italy, Spain and the UK), a substantial proportion of patients with Rome IV confirmed OIC suffered from emotional and psychological symptoms like frustration (28%), feelings of dependency (27%), anxiety (23%), depression (21%) and helplessness (21%) [5]. Among survey participants, 45% reported difficulties in following normal routines, highlighting the importance of adequate OIC handling [5]. Another study reported that patients using opioids with newly diagnosed constipation had higher healthcare utilization and costs than patients without constipation and that these costs accounted for 16% of the healthcare costs per patient [6]. In addition to increased costs, OIC impacts opioid-use [7].

In order to gain better insight into the landscape of opioid therapy in pain patients, anonymized patient data, including demographics, diagnoses, and prescriptions, from the German healthcare data provider “Gesundheitsforen Leipzig” for 2023 were analyzed. The database is validated to be representative of the statutory insured population. Extrapolations indicate that about 4 million patients in Germany received opioids in 2023, among whom 2.3 million received opioids that were potentially OIC-inducing. The fixed combination of tilidine and naloxone accounted for 42% of sales units and was considered less likely to induce relevant OIC. Patients receiving this combination were therefore pragmatically categorized as having a reduced OIC risk within this analysis. Sufentanil, nalbuphine, and meptazinol were likewise not classified as OIC-inducing, given their typical clinical use in short-term or specific care settings. The distribution of patients under potentially OIC-inducing opioid therapy is as shown in Table 1.

Patients on OIC-inducing opioids most frequently received prescriptions for chronic pain; approximately 0.5 million with cancer-related pain and 1.7 million with non-cancer pain. As the majority of opioid-using patients require long-term pain treatment, there is an increased risk of developing persistent OIC. Therefore, increased awareness of the need for adequate counseling and timely management is warranted.

With the development of peripherally acting μ-opioid receptor antagonists (PAMORAs), a treatment for OIC directly targeting opioid receptors in the gut became available, in contrast to conventional laxatives which have a non-specific mode of action. PAMORAs are designed not to cross the blood–brain barrier so that their antagonistic effect is limited to peripheral opioid receptors [8,9]. Clinical studies have demonstrated that PAMORAs effectively treat OIC without interfering with the analgesic effect of opioids [8,9]. To the best of our knowledge, there are not sufficient studies yielding high-level evidence of laxatives being superior for the treatment of OIC so far, as also mentioned by other experts in the field [10]. It should be noted here that economic considerations and local conditions regarding regulatory aspects and availability also play a role in the choice of therapy, as reflected in the individual guidelines. To date, three PAMORAs have been approved for use in Europe: Methylnaltrexone, which is applied subcutaneously; and the two oral PAMORAs naloxegol and naldemedine [9]. In current guidelines the use of PAMORAs is recommended in case of treatment failure with a conventional laxative [1,11,12,13,14,15]. Nevertheless, treatment with laxatives is insufficient in many cases [10,16]. Importantly, OIC and other causes of constipation are frequently coinciding [17]. In these cases, the combination of laxative and PAMORA may be beneficial [17].

To provide healthcare professionals involved in chronic pain management with a comprehensive and practice-oriented aid to improve OIC management, we developed a set of recommendation statements and a treatment escalation algorithm based on international guidelines, additional literature, and the clinical experience of this expert group.

## 2. Materials and Methods

### 2.1. Expert Consensus Process

The aim of this publication was to provide an expert consensus for practical application based on the authors’ expertise and supported by relevant literature. Therefore, the process of consensus building was as described in the following, and not a formal Delphi process.

A literature search for national and international guidelines as well as additional publications on the topics of opioid prescription, the diagnosis of OIC, the first-line therapy of OIC, PAMORA prescription, and the evaluation of PAMORA treatment was performed. For the initial formulation of statements, the following six publications were selected and screened for recommendations on the subjects opioid prescription, the diagnosis of OIC, the first-line therapy of OIC, PAMORA prescription, and the evaluation of PAMORA treatment: Varrassi et al., 2024: Improving Diagnosis and Management of Opioid-Induced Constipation (OIC) in Clinical Practice: An Italian Expert Opinion [1]; Okdahl et al., 2023: Recommendations for the management of opioid-induced constipation—how to improve usability in clinical practice [13]; Ueberall, 2019: DGS-PraxisLeitlinie Opioidinduzierte Obstipation (Version: 2.0 für Fachkreise) [DGS Practice Guideline Opioid-Induced Constipation] [15]; Davies et al., 2024: Inadequate management of opioid-induced constipation in European cancer pain patients: Results of a real-world, multicentre, observational study (“E-StOIC”) [17]; Pohl et al., 2022: Therapeutisches Management der chronischen Obstipation [18]; Luthra et al., 2019: Efficacy of pharmacological therapies for the treatment of opioid-induced constipation: Systematic review and network meta-analysis [19]. The relevant sections of these publications for the abovementioned topics were identified and summarized in 15 initial statements.

On 5 September 2025, the panel of authors from the fields of gastroenterology, pain management, neurology, palliative medicine, and oncology met and the individual statements were discussed. The authors expressed and discussed changes to the initial draft during the live discussion, statements were iteratively adapted until no further changes were desired, and a version was finalized which received at least 90% agreement in an open vote. The results of the voting are noted below the respective statement. During this process, two statements were merged into one, one statement was discarded due to overlap with other statements, and one statement was discarded due to being beyond the scope of this consensus paper, resulting in twelve final statements. With the remaining 12 consensus statements, the manuscript was prepared, then underwent a review by all authors, and the requested changes were made. For each statement, an accompanying explanatory text is provided outlining the rationale and summarizing the supporting literature and/or expert recommendations. A treatment escalation algorithm flow-chart was then derived from the finalized expert consensus statements after the meeting. It should be noted that the conclusions and recommendations of the expert group may be influenced by the inherent limitations of consensus-based methodologies. Although the process was structured and informed by the available literature, expert consensus does not replace systematic evidence synthesis and may be subject to several potential sources of bias, such as personal experiences and opinions on the management of OIC, costs and reimbursement policies, pre-existing national guidelines in their historical context and regulatory aspects of health care utilization in Germany. Nevertheless, in the context of limited evidence, structured expert consensus remains a pragmatic and transparent approach to provide clinical guidance until more robust comparative data become available. As noted in the funding and conflicts of interest sections, the study was funded by Mylan Germany GmbH (a Viatris Company), Bad Homburg, Germany. The funder supported the coordination of the expert board and medical writing. All content-related decisions, including evidence appraisal and the formulation of recommendations, were made independently by the authors.

### 2.2. Healthcare Provider Data Analysis

For the analysis of patient data from Gesundheitsforen Leipzig, the definitions were as follows: Acute pain: A single opioid prescription with a maximum treatment duration of four weeks and no follow-up prescription within that period; multiple acute treatments within one year were counted separately. Tumor patient: Patients with International Classification of Diseases (ICD) code C00-C97 (malignant neoplasms) in the same or up to two quarters before opioid treatment and opioid treatment of one or more opioid prescriptions within four weeks. Palliative care: Patients with ICD code Z51.5 (palliative care) or palliative EBM (German reimbursement code “Einheitlicher Bewertungsmaßstab”) code in same or following quarter of opioid treatment. The data was obtained from billing data from statutory health insurance companies, which are provided in the German Analysis Database for Health Services Research and Evaluation (DADB, Deutsche Analysedatenbank für Versorgungsforschung und Evaluation) and managed by the Gesundheitsforen Leipzig GmbH, Leipzig, Germany. The DADB contains pseudonymized aggregated billing data from 16 statutory health insurance companies with approximately 4.4 million insured persons, constituting a 5% sample of persons with statutory health insurance in Germany. Within this framework, the participating health insurance companies are distributed throughout Germany. The database contains information on master data (age, gender, insured days, etc.), time-related diagnoses, prescriptions and cost information for the main service areas. The reliability und validity of the database is annually verified by the comparison of age-distribution, gender-distribution, morbidity, and mortality with the total population of the statutory health insurance. No statistical differences between the database and total population of the statutory health insurance have been found, so that the sample can be regarded as representative, as also recognized by German regulatory agencies.

## 3. Recommendations

**(1) The clinical entity of opioid-induced constipation (OIC), an aspect of opioid-induced bowel dysfunction (OIBD), is pathophysiologically characterized by peripheral opioid receptor activation, leading to reduced intestinal motility, secretion, and sphincter dysfunction. Moreover, prolonged transit leads to increased liquid absorption, causing dryer and harder stools. It must be differentiated from functional constipation. (Agreement: 13/13)**

In the gastrointestinal tract, delta- (δ), kappa- (κ) and mu- (μ) opioid receptors have been found [3]. The activation of opioid receptors in the intestine has three major effects, namely a change in intestinal motility, decreased secretion, and increased sphincter tone [3]. By stimulating opioid receptors in the enteric nervous system of the myenteric plexus and the submucosal plexus, opioids cause the clinical entity of OIC, which is part of the broader OIBD. Constipation, more specifically OIC, is one symptom of OIBD, in addition to other symptoms, such as abdominal pain, anorexia, bloating, dry mouth, gastro-oesophageal reflux, hard stool consistency, straining, incomplete bowel movements and vomiting [3,20]. Most studies and the following statements are focused primarily on OIC, which is described as the most prevalent and relevant symptom [3]. In contrast to other side effects of opioids, such as sedation, respiratory sedation and nausea, OIC does not typically improve over time [1,2]. Therefore, the handling of OIC is of particular importance for patients undergoing long-term opioid treatment.

**(2) When initiating, adjusting, escalating or switching opioid therapy, especially if treatment is expected to last more than 2–3 weeks, patients should be informed about the risk of OIC and clearly educated on its characteristics in order to prevent the underdiagnosis and undertreatment of OIC. Education includes expected changes in bowel habits, the importance of countermeasures, and when to seek medical advice. (Agreement: 13/13)**

The first barrier in preventing and counteracting OIC is insufficient communication between HCPs and patients on this topic. Patients may be hesitant to inform the physician about constipation, either due to embarrassment or lack of knowledge about the connection to opioids [13]. Therefore, physicians should address this issue proactively at an early stage of opioid treatment [13]. Patients should be educated on OIC when opioid therapy is initiated, modified, escalated, or switched, and if opioid therapy is expected to last longer than 2–3 weeks. Suitable education should help patients recognize the characteristics of OIC and address this topic during consultations. Importantly, it is advised that patients are also asked about signs of OIC during the course of opioid treatment at follow-up appointments [13]. Furthermore, patients should be educated that not only reduced frequency of bowel movements is a sign of OIC but, in the broader context of OIBD, additional symptoms may occur. These measures may help prevent patients from performing self-withdrawal or opioid dose reduction, which in turn can compromise effective pain management [1].

**(3) Systematic OIC screening and structured documentation should be integrated into routine clinical workflows. Patients should be evaluated for constipation prior to the start of opioid therapy and no later than the second week after starting opioid therapy, with one of the established tools, such as the “Bowel Function Index” (BFI). Stool frequency and stool texture should be assessed. (Agreement: 13/13)**

**Figure 1:**
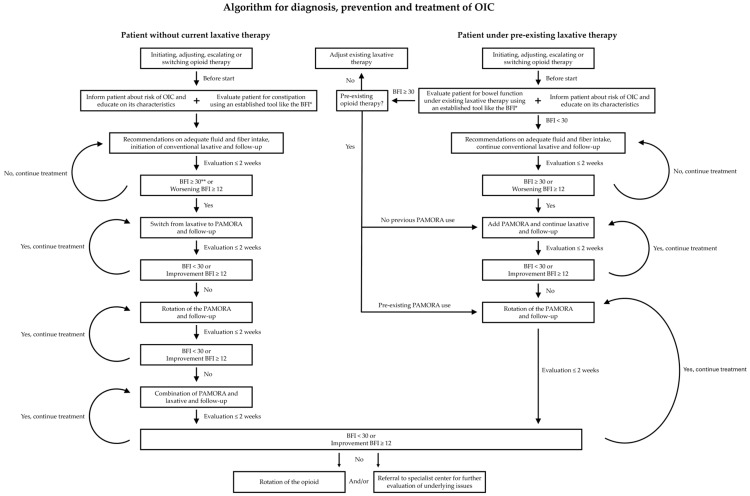
Algorithm for diagnosis, prevention, and treatment of OIC. The algorithm summarizes the recommendations described in the main text, differentiating between patients without laxative treatment prior to the start of opioid therapy and patients under pre-existing laxative therapy. * BFI: Patients rate three items according to their experience in the past 7 days on a scale from 0 to 100 (100 = most severe). (1) Ease of defecation; (2) Feeling of incomplete bowel evacuation; (3) Personal judgment of constipation. ** For practical reasons, the authors agreed to use a BFI score of ≥ 30 as indicative of OIC. This threshold was therefore chosen as the reference value. This approach is consistent with prior studies and consensus publications. OIC: Opioid-Induced Constipation; BFI: Bowel Function Index; PAMORA: Peripherally acting μ-opioid receptor antagonist. The algorithm reflects current evidence and expert opinion but has not yet undergone prospective validation.

Patients should be evaluated for constipation before starting opioid therapy to document baseline gastrointestinal function [13]. A follow-up assessment for constipation should be performed no later than the second week after starting opioid therapy and the results of both assessments should be compared in order to evaluate the possible impact of opioid therapy on gastrointestinal function [1]. A suitable tool, validated for opioid-treated patients with pain, is the BFI [21]. This tool consists of three variables: “ease of defecation”, “feeling of incomplete bowel evacuation”, and “personal judgment of constipation”. These variables are rated by the patient on a numerical analog scale (NAS) of 0–100 with 0 representing freedom from the symptom and 100 representing highest severity [21]. BFI values between 0 and 28.8 were observed in 95% of non-constipated chronic pain patients, defining this interval as the reference range for non-constipated patients [21]. For practical reasons, the authors agreed to use a BFI score of ≥30 as indicative of OIC (9 approvals, 1 rejection, 3 abstentions). This threshold was therefore chosen as the reference value in the accompanying algorithm (Figure 1). This approach is consistent with prior studies and consensus publications [1,22,23].

In addition to the BFI, Rome IV criteria have been defined for OIC [17]:

1. New, or worsening symptoms of constipation when initiating, changing, or increasing opioid therapy, that must include two or more of the following:
  - Straining during more than 25% of defecations
  - Lumpy or hard stools (Bristol Stool Form Scale 1–2) in more than 25% of defecations
  - Sensation of incomplete evacuation during more than 25% of defecations
  - Sensation of anorectal obstruction/blockage during more than 25% of defecations
  - Manual maneuvers to facilitate more than 25% of defecations
  - Fewer than three spontaneous bowel movements (SBM) per week.
2. The patient must meet the following criterion: Loose stools are rarely present without the use of laxatives.

A significant association between the BFI and Rome IV criteria was demonstrated in a recent study, confirming the appropriateness of both tools for clinical use [17]. In the same study, it was shown that simply asking the patients if they are constipated is not sufficient and a more thorough assessment is required [17]. While the Rome IV criteria is in principle suitable for the diagnosis of OIC, the authors of this publication agree that the use of the BFI is recommended for clinical practice.

Additionally, based on their expertise, the authors recommend that the stool texture is evaluated. A suitable tool for this assessment is the Bristol Stool Scale, which categorizes the stool form on a 7-point scale and has been shown to be useful for monitoring changes in intestinal function [24]. The application of established tools for the evaluation of OIC, as described above, can contribute to timely and adequate intervention [13]. Other potential contributors for constipation, including medication and comorbidities, should be assessed and excluded [13]. Due to the substantial impact of OIC on quality of life, patient well-being can be assessed using the Patient Assessment of Constipation Quality of Life questionnaire (PAC-QOL) [17,25]. It comprises subscales for psychosocial discomfort, worries, concerns and overall satisfaction [17,25].

**(4) Due to the known multifactoriality of constipation, patients should receive recommendations for prevention, including adequate fluid and fiber intake, and physical activity if possible. (Agreement: 13/13)**

Generally, in the context of patient education, basic measures for preventing constipation should be recommended. These include sufficient fluid and fiber intake as well as physical activity [13,17]. For some patients, particularly in cases of advanced cancer, these interventions can be difficult [17]. Therefore, individualized management of constipation is important.

**(5) Due to the complexity of constipation, standard laxatives, particularly macrogol or a stepwise combination of stimulant and osmotic agents, should be initiated early, either alongside the opioid (especially for high-risk patients with immobilization, etc.) or at the first sign of constipation. If constipation persists despite the correct use of the laxative, patients should contact their physician, and the treatment should be reviewed, and the use of PAMORAs (peripherally acting μ-opioid receptor antagonists) should be evaluated. (Agreement: 12/13; 1 abstention)**

Before initiating opioid therapy, patients should be systematically screened for constipation risk factors. A prior history of constipation is the most important predictor of OIC [26]. Additional risk factors include female sex, low physical activity, low fluid intake, comorbidities, certain surgeries (especially gynecological, abdominal and anorectal) and the use of constipating medications [27]. Particularly in patients with cancer, various organic, functional, and pharmacological circumstances can lead to a risk for constipation [28].

Laxative therapy should be started simultaneously to the opioid treatment or not later than the first sign of constipation [13]. The decision to start prophylactic laxative use should be made on a case-dependent basis [1]. A recent study found that a substantial number of patients (33%), who were prescribed a laxative or PAMORA, did not take this medication on a daily basis [17]. This finding demonstrates that patients should be educated on the importance of daily application.

The use of laxatives as first-line therapy is supported by indirect evidence, clinical experience, and endorsement in current guidelines. However, high-quality data from randomized controlled trials specifically designed to evaluate laxatives in OIC remain limited [10]. In a retrospective study in the USA, Canada, Germany, and the UK with patients with chronic non-cancer pain, it was found that the vast majority (94%) of patients, who took appropriate doses of a laxative, had an inadequate response [10,16]. Therefore, in most cases, treatment of OIC with a laxative alone is probably not sufficient. The authors of the publication presented here agree that from a mechanistic point of view, the treatment of OIC with PAMORAs as first-line therapy would appear as reasonable. However, due to economic reasons and the design of the approval studies for PAMORAs as second line treatments, laxatives remain the first-line treatments according to relevant guidelines. Since the treatment algorithm (Figure 1) is designed for practical use primarily in Germany considering the German reimbursement regulations, laxatives are therefore mentioned as first treatment option, following national guidelines [11,12,29].

**(6) Osmotic or stimulant laxatives are preferred over non-absorbable sugars, which may worsen bloating due to fermentation. Polyethylene glycol-(PEG) based products are often necessary. (Agreement: 13/13)**

Fiber-based bulking laxatives may worsen abdominal distention, so that these agents are generally not appropriate for the prevention or treatment of OIC [1]. Osmotic laxatives can be used in order to increase the water content of the stool, softening the stool consistency and facilitating bowel movements. PEG belongs to the group of osmotic laxatives and has been described as a favorable first-line agent in OIC due to its safety profile and stool-hydrating effect [1]. Against the background of a paucity of studies, a placebo-controlled trial of PEG and lactulose for treatment of OIC at a methadone maintenance program is mentioned, revealing a PEG treatment as more effective for achieving loose stools than lactulose or placebo with less adverse effects than lactulose [30]. These results were confirmed by a prospective, open-label investigation of PEG, sodium picosulphate, and lactulose in ambulatory cancer patients on opioid therapy, which found PEG or sodium picosulphate to be more effective than lactulose [31]. A major concern with the use of non-absorbable sugars like lactulose is that they can induce bloating and abdominal distension [32]. Therefore, non-absorbable sugars are not recommended for the treatment of OIC [13]. Stimulant laxatives may be used as first-line treatment for OIC, but their effectiveness has not been proven by clinical trials [1].

**(7) After treatment with a laxative, the effect should be evaluated within a time frame of two weeks. If constipation persists, the treatment with a PAMORA should be initiated. (Agreement: 13/13)**

According to the Italian Medicine Agency, resistance to laxatives is defined as “lack of response after three days”. Therefore, the authors of the Italian expert opinion recommend that patients should contact the physician for a review of therapy after this time frame [1]. According to the German DGS (Deutsche Gesellschaft für Schmerzmedizin e.V.) Practice Guideline Opioid-Induced Constipation, treatment failure occurs if no treatment success can be measured after 1–2 weeks [15]. In a number of German and international guidelines, initiation of a PAMORA therapy is recommended if laxatives are not sufficiently effective [1,11,12,13,14,15,29]. In order to evaluate treatment success, use of the BFI is recommended [1,15,29]. For monitoring changes in the intestinal function due to opioid use, the BFI should be compared to the BFI assessment immediately before the start of opioid therapy. The authors recommend a time frame of two weeks as practical and reasonable to assess the success of laxative treatment. This time frame also follows recently published treatment recommendations, where evaluation of bowel function within 2 weeks of starting opioids is advised in the “Seven-step clinical pathway for effective management of OIC” [23]. The authors stress that early assessment of bowel function is essential and in case of laxative treatment failure, PAMORA treatment should be initiated [23].

**(8) A stepwise approach with conventional laxatives followed by a PAMORA should be used for management of OIC. In cases of insufficient response, several escalation strategies may be considered, including rotation of the PAMORA, a combination of laxatives and PAMORA, or pursuing alternative interventions. The decision to escalate should be based upon biweekly clinical follow-ups. Using BFI scores for follow-ups is recommended. Rotation of the PAMORA before a combination of laxative and PAMORA is recommended based on the authors’ expertise, but it should be noted that currently there is no direct scientific evidence for the benefits of PAMORA rotation. (Agreement: 13/13)**

A stepwise approach, guided by the BFI, has already been successfully applied in a feasibility study [22]. The authors therefore agree that use of the BFI as a tool validated for patients with pain is recommended in order to guide treatment decisions with the steps described above and in the treatment algorithm (Figure 1). The proposed treatment algorithm represents a synthesis of available evidence and expert opinion. However, it has not yet undergone prospective validation, and its clinical utility should therefore be confirmed in future studies.

Similar stepwise approaches have been published [1,11,13,20]. The option of the rotation of the PAMORA prior to initiating combination therapy is based on the clinical experience of the expert panel rather than on direct evidence. While randomized studies evaluating PAMORA rotation are lacking, the rationale for this approach lies in the potential interindividual differences in treatment response and tolerability. The rotation of opioids is an established strategy in routine clinical practice, commonly employed to optimize analgesic efficacy and improve tolerability where required [33]. However, based on their experience, the experts of this panel view opioid rotation as more burdensome for patients than PAMORA rotation, as it involves changes to the analgesic regimen with potential impacts on pain control. Prevention or the early treatment of OIC may improve tolerability and reduce gastrointestinal side effects [34]. Therefore, the authors of the work presented here recommend monitoring gastrointestinal function in intervals of two weeks and adjusting therapy according to the stepwise protocol until the BFI indicates the absence of OIC (BFI < 30).

Still, the experts recommend considering the needs of individual patients. Therefore, the two-week interval represents a general timeframe rather than a strict rule, and earlier evaluation is appropriate in symptomatic or high-risk individuals. Following this approach, in patients with advanced disease, high symptom burden, or limited life expectancy, particularly in palliative care settings, laxative and PAMORA therapy should be guided by individual risk profiles and patient priorities rather than rigid escalation protocols. Since the BFI is not applicable for all patient groups, for example during intensive care, other assessments, such as stool frequency and stool texture, can also be used.

**(9) PAMORAs are effective in treating OIC, and treatment with PAMORAs should be initiated in cases of non-respondence or intolerance to at least one laxative. Patients should be educated on the need to take the PAMORA regularly, not on demand. In cases of treatment failure, poor adherence to laxatives or PAMORA therapy should be routinely assessed. (Agreement: 13/13)**

Various studies have demonstrated the efficacy and safety of PAMORAs for the treatment of OIC [1,11,12,13,15]. Despite the availability of effective and well-tolerated PAMORAs, OIC continues to be underdiagnosed and insufficiently treated [1,13].

PAMORAs are molecules that directly bind to peripheral opioid-receptors as antagonists and inhibit their activation by opioids [8,9,19]. By targeting peripheral receptor-mediated effects in the gastrointestinal tract, PAMORAs address the key pathophysiological mechanism of OIC. In contrast, conventional laxatives exert their effects through non-receptor-mediated mechanisms. PAMORAs are designed not to cross the blood–brain barrier to a clinically relevant extent, so that the analgesic effect of opioids is not compromised [8,9]. As with laxative therapy, patients should be educated about the importance of taking PAMORAs daily. During follow-up visits, adherence to treatment should be assessed if the treatment response is inadequate.

**(10) Before prescribing a PAMORA, potential drug interactions and contraindications such as gastrointestinal stenosis must be reviewed. It is recommended to discontinue laxative use with the start of PAMORA therapy if the patient did not use laxatives before starting opioid therapy. (Agreement: 13/13)**

Gastrointestinal obstruction or the risk of the recurrence of a gastrointestinal obstruction are contraindications for the use of PAMORAs due to the risk of gastrointestinal perforation [9]. Therefore, patients need to be reviewed for gastrointestinal stenosis before therapy initiation [13].

Naloxegol and naldemedine are substrates of cytochrome P450 3A4 (CYP3A4) and P-glycoprotein, hence possible drug interactions should be ruled out [1,35]. The induction or inhibition of these pathways may lead to decreased or increased PAMORA-levels, respectively [35]. In contrast, methylnaltrexone does not interact with CYP isozymes to a relevant extent [1,35]. The use of PAMORAs together with other opioid antagonists or partial agonists in patients who also receive a full agonist opioid may reduce the analgesic effect or promote withdrawal symptoms [1,9].

**(11) In some patients, OIC coexists with other causes of constipation. Therefore, the combined use of a PAMORA and conventional laxatives may be necessary, especially in patients who had constipation before and were on laxatives prior to opioid use, each targeting different mechanisms. (Agreement: 13/13)**

Many patients suffering from OIC simultaneously suffer from other causes of constipation [17,36]. Therefore, it may be necessary to treat these patients with a laxative and a PAMORA simultaneously. In these cases, the PAMORA targets the OIC while the laxative manages other causes of constipation [17].

Following this consideration, as illustrated in the depicted algorithm (Figure 1), after the initiation of PAMORA therapy, laxatives can be reintroduced, depending on personal requirements and treatment success [23]. If the patient received laxative treatment already before the start of opioid treatment, the authors of this consensus paper recommend that the PAMORA should be introduced in addition to the laxative, when signs of OIC occur (Figure 1).

**(12) If PAMORA response remains inadequate after two weeks, options include: Combination with laxatives; rotation of the opioid; referral to a specialist center where further evaluation of underlying issues should be made. Diagnostic work-up should include clinical reassessment, laboratory tests, and imaging as needed, and may involve multidisciplinary consultation. (Agreement: 13/13)**

If PAMORA treatment does not lead to satisfactory results, further efforts to provide symptom relief should be made. First, as described in the previous section, a laxative can be introduced additionally, so that OIC and other causes of constipation are addressed simultaneously. If no treatment success is achieved with this combination therapy, a rotation to a different opioid should be considered. Opioid rotation may alter the balance between analgesia and adverse effects, so that it can be a strategy to overcome opioid-related adverse effects, such as OIC [1]. Fixed combinations of opioid agonist and antagonist (for example oxycodone and naloxone) are linked to a lower risk of constipation as well [37]. If the treatment of OIC fails despite this multi-step approach, the patient should be referred to a specialist for gastrointestinal disorders in order to further investigate underlying conditions, comorbidities, and comedication, as also recommended in previous publications [1,13]. It should also be noted that the efficacy of electroacupuncture for the treatment of OIC has been demonstrated with a good safety profile of the treatment [38].

## 4. Conclusions

OIC is a frequent adverse effect of opioid treatment with a significant impact on the quality of life. Nevertheless, it is still underdiagnosed and undertreated. To improve OIC management, twelve statements were developed and adopted based on current publications and guidelines. These statements and the accompanying remarks cover topics of definition, patient education, prevention, diagnosis, treatment, and follow-up examinations. For the individual statements, the strength of evidence varies and the respective basis, meaning published evidence, guidelines and/or authors’ expertise, is described transparently in the accompanying section. These statements are intended to support physicians and other healthcare professionals in the detection and adequate management of OIC. This publication includes an algorithm for the diagnosis and treatment of OIC for daily practice. The algorithm outlines a stepwise management approach that considers prior laxative use and recommends evaluating the treatment response every two weeks using the BFI. Overall, the recommendations highlight the importance of early OIC management and systematic monitoring with a suitable validated tool. The stepwise treatment scheme starts with laxatives and then proceeds to PAMORA therapy, following current guidelines. PAMORAs represent a mechanism-oriented option for the treatment of OIC and have demonstrated efficacy in randomized controlled trials. Accordingly, they are recommended in patients with an inadequate response to conventional laxatives. Importantly, the international generalizability of the proposed recommendations may be limited by economic considerations, differences in healthcare systems, regulatory environments, drug availability, and reimbursement policies. Consequently, adaptation to local clinical practice and national regulations may be required. This publication aims to increase awareness of OIC, so that it is proactively addressed as an integral part of opioid therapy initiation and management. This approach aims to achieve timely and adequate management of OIC which ultimately contributes to enhanced quality of life.

## Figures and Tables

**Table 1:** Distribution of patients under potentially OIC-inducing opioids. Patient data from the validated, representative German healthcare database “Deutsche Analysedatenbank für Versorgungsforschung und Evaluation” (DADB) of 4.4 million patients with statutory health insurance were extrapolated to the German population. Data is from 2023. For definitions of the patient groups, see methods section.

Patient Data from DADB	
**Patients on OIC-inducing opioids**	2,326,416
**Acute pain (≤4 weeks opioids)**	127,936
**Chronic cancer pain**	542,509
Palliative care	175,021
Non-palliative care	367,488
**Chronic non-cancer pain**	1,655,971
Palliative care	116,754
Non-palliative care	1,539,216

## Abbreviations

The following abbreviations are used in this manuscript:

BFI	Bowel Function Index
CYP3A4	cytochrome P450 3A4
DADB	Deutsche Analysedatenbank für Versorgungsforschung und Evaluation
DGS	Deutsche Gesellschaft für Schmerzmedizin e.V.
EBM	German reimbursement code “Einheitlicher Bewertungsmaßstab”
HCP	healthcare professional
ICD	International Classification of Diseases
NAS	numerical analog scale
OIBD	opioid-induced bowel dysfunction
OIC	opioid-induced constipation
PAC-QOL	Patient Assessment of Constipation Quality of Life questionnaire
PAMORA	peripherally acting μ-opioid receptor antagonist
PEG	polyethylene glycol
SBM	spontaneous bowel movement
